# Vitamin D Status and Its Association With Disease Severity in Hashimoto’s Thyroiditis

**DOI:** 10.7759/cureus.83419

**Published:** 2025-05-03

**Authors:** Eligio Copari-Vargas, Tania Libertad Copari-Vargas, Luis Fernando Domínguez-Valdez, Luisa Elena Copari-Vargas, Eligio Copari-Jimenez

**Affiliations:** 1 Endocrinology, Diabetes, and Metabolism, Hospital General de México "Dr. Eduardo Liceaga", Mexico, MEX; 2 Internal Medicine, Hospital de Clínicas, La Paz, BOL; 3 Internal Medicine, Unidad Médica de Alta Especialidad (UMAE) Hospital de Especialidades "Dr. Antonio Fraga Mouret" del Centro Médico Nacional La Raza, Mexico, MEX

**Keywords:** anti-thyroglobulin antibodies, thyroid autoimmunity, vitamin d, vitamin d deficiency, hashimoto’s thyroiditis

## Abstract

Introduction

Hashimoto’s thyroiditis (HT), the most common autoimmune thyroid disorder, involves chronic lymphocytic infiltration and thyroid dysfunction. Vitamin D deficiency has been linked to autoimmune thyroid diseases, suggesting an immunomodulatory role. This study examines the association between serum vitamin D levels and HT severity in patients at a secondary-level hospital in Mexico.

Methods

A retrospective observational study was conducted on adult patients diagnosed with HT from January 2022 to January 2024. Clinical and laboratory data, including serum 25-hydroxyvitamin D (25(OH)D), thyroid-stimulating hormone (TSH), free thyroxine (FT4), and anti-thyroid peroxidase (anti-TPO) antibodies, were collected. Patients were classified by thyroid function and vitamin D status. Statistical analyses were performed to evaluate correlations and group associations to determine the relationship between vitamin D deficiency and HT severity.

Results

A total of 114 patients were included in the study: 1.8% (n=2) were classified as euthyroid, 18.4% (n=21) as having subclinical hypothyroidism, and 79.8% (n=91) as having overt hypothyroidism. Vitamin D deficiency, defined as serum 25(OH)D levels below 20 ng/mL, was highly prevalent, affecting 49.5% (n=45) of patients with overt hypothyroidism and 42.9% (n=9) of those with subclinical hypothyroidism. A significant inverse correlation was observed between 25(OH)D levels and anti-TPO antibody titers (p=0.01), suggesting an association between lower vitamin D concentrations and increased autoimmune activity. Additionally, vitamin D-deficient patients exhibited significantly higher TSH levels compared to those with sufficient vitamin D (p=0.01). No significant differences were found in FT4 levels across the different vitamin D categories.

Conclusions

This study suggests an inverse correlation between vitamin D deficiency and the severity of HT, supporting the potential immunomodulatory role of vitamin D. However, due to the observational nature of the study, causal relationships cannot be inferred. Given the high prevalence of vitamin D deficiency among patients with HT, further research is warranted to explore the therapeutic implications of vitamin D supplementation on disease progression and management.

## Introduction

Primary hypothyroidism affects 0.3% to 3.7% of the population in the United States and 0.2% to 5.3% in Europe [1,2]. In Mexico, the estimated prevalence of hypothyroidism is approximately 1% in the general population, with subclinical hypothyroidism occurring in 3% to 8% of individuals [3]. While iodine deficiency remains the leading cause of hypothyroidism worldwide, Hashimoto’s thyroiditis (HT) is the most common organ-specific autoimmune disorder. HT results from a complex interplay between genetic predisposition and environmental triggers, including iodine and selenium imbalances, certain medications, irradiation, smoking, infections, and psychological stress, and is characterized by lymphocytic infiltration into the thyroid gland along with the production of thyroid-specific autoantibodies [4,5]. Beyond its role in bone metabolism, vitamin D (Vit D) exerts immunomodulatory effects, including inhibition of pro-inflammatory cytokines, regulation of Th1 and Th17 lymphocytes, and suppression of B-cell differentiation [6]. Serum 25-hydroxyvitamin D (25(OH)D) levels serve as an integrated marker of systemic vitamin D status, reflecting both cutaneous synthesis and dietary intake [7,8]. This study aims to assess the prevalence of vitamin D abnormalities in patients with chronic autoimmune thyroiditis by examining the association between vitamin D levels and autoimmune thyroid disease.

## Materials and methods

Between January 2024 and January 2025, patients attending the outpatient endocrinology clinic at Hospital General de México “Dr. Eduardo Liceaga” were screened for inclusion. Clinical records from January 2022 to January 2024 were reviewed. Inclusion criteria consisted of adults (≥18 years) with confirmed chronic autoimmune thyroiditis and available data on anti-thyroid peroxidase (anti-TPO) and 25(OH)D levels. Exclusion criteria included postmenopausal women, smokers, and patients receiving thyroid-altering medications (e.g., lithium, amiodarone, steroids, beta-blockers, interferon), diuretics, or contraceptives. Patients with renal or hepatic failure, heart failure, malnutrition, malignancy, pregnancy, or other endocrine disorders were also excluded.

The sample size was calculated based on an estimated prevalence of HT of 8.6%, a 95% confidence level, and a precision of 0.05, yielding a minimum required sample of 114 cases. Quantitative variables included age, weight, anti-TPO antibody levels (IU/mL), thyroid-stimulating hormone (TSH) levels (mIU/L), and free thyroxine (FT4) levels (ng/dL). Categorical variables included sex and BMI.

Patients were classified based on 25(OH)D serum concentrations as follows: severe deficiency (<5 ng/mL), mild deficiency (5-20 ng/mL), insufficiency (21-30 ng/mL), and sufficiency (>30 ng/mL). Subclinical hypothyroidism was defined as elevated TSH levels (>4.5 mIU/L) with normal FT4 concentrations. Anti-TPO antibody titers were categorized as mild (35-100 IU/mL), moderate (101-200 IU/mL), or severe (>200 IU/mL), according to established clinical thresholds. The study protocol was reviewed and approved by the research committee of Hospital General de México “Dr. Eduardo Liceaga,” with registration number DECS/JPO-CT-2278-2024. Minor modifications were suggested and implemented before data collection commenced.

Analyses were performed using IBM SPSS Statistics for Windows, Version 25.0 (IBM Corp., Armonk, NY, USA). Categorical variables were expressed as frequencies and percentages; continuous variables as means ± SDs. Chi-square tests were used for categorical comparisons, and independent t-tests for continuous variables. Vitamin D status was categorized as >30 ng/mL (sufficient), 20-30 ng/mL (insufficient), and <20 ng/mL (deficient). Correlations between 25(OH)D, TSH, and anti-TPO levels were analyzed. A p-value <0.05 was considered significant.

## Results

A total of 114 patients were included in the study: 1.8% (n=2) were classified as euthyroid, 18.4% (n=21) had subclinical hypothyroidism, and 79.8% (n=91) presented with overt hypothyroidism. The mean age was 45.8 ± 13.5 years, with no significant differences among groups (p=0.220). Female patients accounted for 61.4% (n=73) of the total, with a higher proportion in the overt hypothyroidism group at 67.0% (n=61), compared to 52.4% (n=11) in the subclinical group and 50.0% (n=1) in the euthyroid group (p=0.414). The mean BMI was 28.7 ± 6.1 kg/m², showing no significant differences among groups (p=0.350). Comorbidities were reported in 26.3% (n=30) of patients overall, occurring more frequently in the overt hypothyroidism group at 29.7% (n=27), compared to 9.5% (n=2) in the subclinical group (p=0.125). The most common comorbidities included type 2 diabetes mellitus (22.8%) and hypertension (7.0%), with isolated cases of alcohol use disorder and tobacco use (each accounting for 0.9%). Autoimmune diseases were identified in 24.6% (n=28) of patients, with no statistically significant difference between groups (p=0.561). The most frequently reported autoimmune conditions were rheumatoid arthritis (5.3%), systemic lupus erythematosus (4.4%), and Sjögren’s syndrome (5.3%), followed by isolated cases of systemic sclerosis (1.8%), autoimmune hepatitis (1.8%), Addison’s disease (2.6%), and pernicious anemia (0.9%).

Thyroid nodules were significantly more prevalent in patients with overt hypothyroidism at 11.0% (n=10), while no cases were reported in the subclinical or euthyroid groups (p=0.038). Goiter was detected in 21.9% (n=25) of the patients, with the highest prevalence in the overt hypothyroidism group at 24.2% (n=22) (p=0.635).

Regarding the lipid profile, low-density lipoprotein cholesterol (LDL-C) levels were found to be significantly lower in hypothyroid patients (p=0.048), an observation that differs from the lipid alterations typically described in hypothyroidism. No significant differences were detected in total cholesterol, triglycerides, or high-density lipoprotein cholesterol (HDL-C) levels among groups. Thyroid function markers, including TSH and FT4, showed no significant differences between groups (p=0.464 and p=0.912, respectively). Vitamin D levels were similar across groups (p=0.954), with most patients exhibiting concentrations below the optimal threshold. Vitamin D deficiency (<20 ng/mL) was observed in 49.5% (n=45) of patients with overt hypothyroidism and 42.9% (n=9) of those with subclinical hypothyroidism (p=0.965) (Table 1).

**Table 1:** Demographic profile. TG: Triglycerides; TC: Total Cholesterol; HDL-C: High-Density Lipoprotein Cholesterol; LDL-C: Low-Density Lipoprotein Cholesterol; TSH: Thyroid-Stimulating Hormone; FT4: Free Thyroxine; Anti-TPO: Anti-Thyroid Peroxidase Antibodies; 25(OH)D: 25-Hydroxyvitamin D; N/A: Not Applicable.

Character	Euthyroidism (n=2)	Subclinical Hypothyroidism (n=21)	Overt Hypothyroidism (n=91)	P-value
Age, years (mean ± SD)	42.0 ± 2.8	41.5 ± 13.5	47.1 ± 13.6	0.22
Sex, n (%)	N/A	N/A	N/A	0.414
Female	1 (50.0%)	11 (52.4%)	61 (67.0%)	N/A
Male	1 (50.0%)	10 (47.6%)	30 (33.0%)	N/A
Height, m (mean ± SD)	1.5 ± 0.0	1.5 ± 0.6	1.55 ± 0.0	0.354
Weight, kg (mean ± SD)	84.8 ± 0.2	67.6 ± 12.7	67.2 ± 12.6	0.167
BMI, kg/m² (mean ± SD)	36.2 ± 1.8	27.2 ± 6.1	27.6 ± 4.7	0.35
Presence of comorbidities, n (%)	1 (50.0%)	2 (9.5%)	27 (29.7%)	0.125
Presence of autoimmune disease, n (%)	0 (0.0%)	5 (23.8%)	23 (25.3%)	0.561
Goiter, n (%)	0 (0.0%)	3 (14.3%)	22 (24.2%)	0.635
Thyroid nodules, n (%)	1 (50.0%)	0 (0.0%)	10 (11.0%)	0.038
TG, mg/dL (mean ± SD)	188.5 ± 51.6	138.2 ± 78.3	162.9 ± 80.7	0.39
TC, mg/dL (mean ± SD)	238.5 ± 4.9	174.7 ± 51.8	178.8 ± 40.5	0.133
HDL-C, mg/dL (mean ± SD)	50.5 ± 3.5	46.0 ± 15.5	48.9 ± 18.8	0.793
LDL-C, mg/dL (mean ± SD)	163.0 ± 2.8	111.4 ± 41.0	111.0 ± 30.2	0.048
TSH, mIU/L (mean ± SD)	3.0 ± 0.7	5.4 ± 2.7	9.9 ± 18.1	0.464
FT4, ng/dL (mean ± SD)	1.3 ± 0.1	0.9 ± 0.1	1.0 ± 1.3	0.912
Anti-TPO, U/mL (mean ± SD)	284.0 ± 118.7	947.8 ± 983.8	566.9 ± 765.5	0.128
25(OH)D, ng/mL (mean ± SD)	19.8 ± 5.8	20.9 ± 6.4	20.6 ± 5.9	0.954
25(OH)D classification, n (%)	N/A	N/A	N/A	0.965
>30 ng/mL	0 (0.0%)	1 (4.8%)	5 (5.5%)	N/A
20-30 ng/mL	1 (50.0%)	11 (52.4%)	41 (45.1%)	N/A
<20 ng/mL	1 (50.0%)	9 (42.9%)	45 (49.5%)	N/A

Patients were stratified based on 25(OH)D levels into three categories: >30 ng/mL, 20-30 ng/mL, and <20 ng/mL. A significant inverse relationship was observed between TSH levels and vitamin D status. Patients with vitamin D deficiency (<20 ng/mL) exhibited the highest TSH levels (11.5 ± 22.0 mIU/L), compared to those with insufficiency (6.9 ± 7.9 mIU/L, p=0.01) and sufficiency (4.5 ± 1.9 mIU/L, p=0.01). No significant differences were found in FT4 levels, which remained stable across all vitamin D categories (p=0.44-0.93). Anti-TPO levels were markedly elevated in vitamin D-deficient patients (731.8 ± 991.0 U/mL) compared to the sufficient group (539.1 ± 313.4 U/mL, p=0.01), suggesting a potential association between low vitamin D levels and increased autoimmune thyroid activity (Table 2).

**Table 2:** Characteristics of subjects stratified according to 25(OH)D classification. 25(OH)D: 25-Hydroxyvitamin D; TSH: Thyroid-Stimulating Hormone; FT4: Free Thyroxine; Anti-TPO: Anti-Thyroid Peroxidase Antibodies.

25(OH) D classification	TSH		FT4		Anti-TPO	
	Mean (SD)	P-value	Mean (SD)	P-value	Mean (SD)	P-value
>30 ng/mL	4.5 (1.9)	0.01	0.8 (0.1)	0.44	539.1 (313.4)	0.53
20–30 ng/mL	6.9 (7.9)	0.01	1.0 (1.2)	0.7	539.1 (624.2)	0.25
<20 ng/mL	11.5 (22.0)	<0.01	0.9 (1.0)	0.93	731.8 (991.0)	0.01

Correlation between serum 25(OH)D and TSH levels

**Figure 1:**
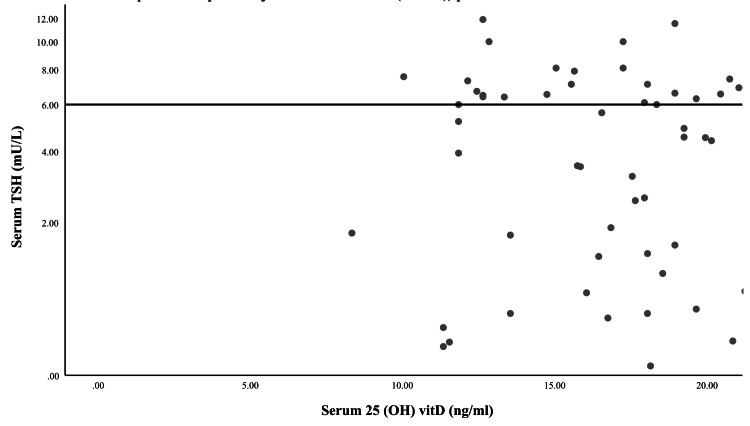
Correlation between 25(OH)D and serum TSH levels in hypothyroid patients. 25(OH)D: 25-Hydroxyvitamin D; TSH: Thyroid-stimulating hormone.

Serum TSH levels showed a clustered distribution, with most values concentrated between 2 and 6 mU/L, while serum 25(OH)D levels were primarily distributed between 5 and 20 ng/mL. A dispersion of data points was observed across the vitamin D spectrum, with some outliers present at higher TSH values. Despite this variability, a subtle trend of decreasing TSH levels with increasing vitamin D concentrations was noted. The heterogeneous distribution of TSH levels may be influenced by individual differences in thyroid function, metabolic status, or treatment regimens, which were not fully accounted for in this dataset. Additionally, the narrow range of vitamin D values in the cohort may contribute to the observed data pattern. While no clear separation of groups was detected, the distribution of values suggests potential underlying physiological interactions that warrant further investigation in a larger and more controlled sample (Figure 1).

Correlation between serum 25(OH)D and free T4 levels

**Figure 2:**
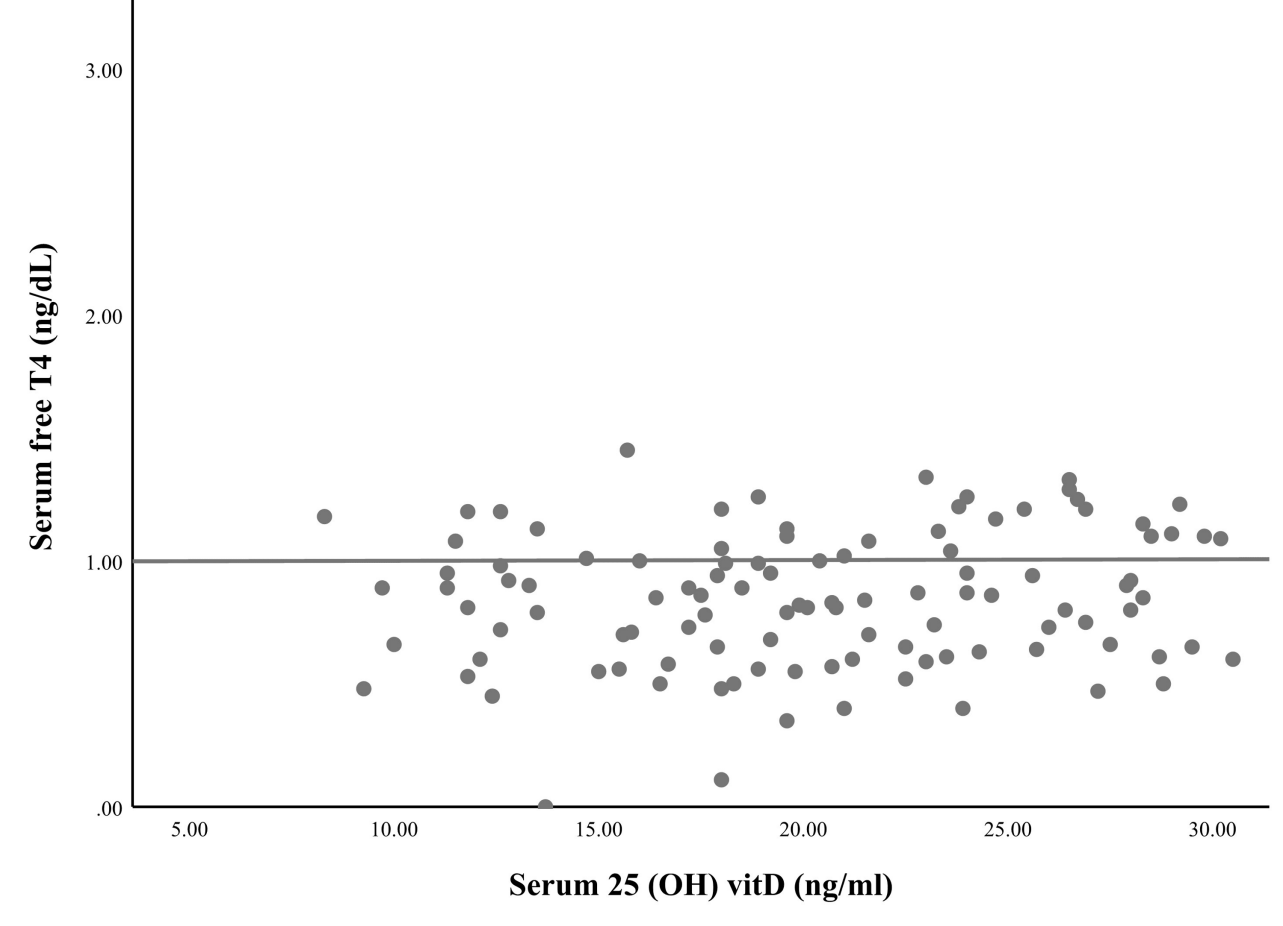
Correlation between 25(OH)D and serum FT4 levels in hypothyroid patients. 25(OH)D: 25-Hydroxyvitamin D; FT4: Free thyroxine.

Serum free T4 levels exhibited a relatively homogeneous distribution, with most values clustering around 1.0 ng/dL, while serum 25(OH)D levels ranged between 5 and 30 ng/mL. The data points were widely dispersed, with no clear separation between groups. The majority of free T4 values remained within a narrow range, with only a few outliers exceeding 2.0 ng/dL. A slight upward trend in free T4 values with increasing vitamin D levels was observed; however, the distribution of data suggests a high degree of variability among participants. The relatively uniform dispersion of free T4 values, despite variations in vitamin D levels, may reflect individual differences in thyroid regulation, compensatory mechanisms, or other confounding metabolic factors that influence thyroid hormone homeostasis. While no strong pattern was evident, the absence of extreme fluctuations in free T4 across different vitamin D levels indicates a stable hormonal profile in the study population, requiring further analysis in more controlled settings to determine potential modulatory effects (Figure 2).

## Discussion

This study examined the association between thyroid function markers, TSH, FT4, anti-TPO, and serum 25(OH)D levels in patients with euthyroidism, subclinical hypothyroidism, and overt hypothyroidism. The findings support previous reports of an inverse relationship between vitamin D status and autoimmune thyroid activity, as first described by Tamer G et al. [7].

Mechanistic insights proposed by Zhao R et al. suggest that vitamin D may exert protective effects against HT through four key pathways: inhibition of T-cell activation via dendritic cell modulation, suppression of HLA class II gene expression in thyroid tissue, regulation of B-cell function, and restoration of the Th17/Treg cell balance [8]. These immunomodulatory functions provide a plausible biological rationale for the observed association between vitamin D deficiency and heightened thyroid autoimmunity [7-10]. In this study, a marked deficiency of 25(OH)D was observed in patients with elevated anti-TPO levels compared to those with adequate vitamin D, reinforcing the potential role of vitamin D deficiency as a contributing factor in the pathogenesis and severity of chronic autoimmune thyroiditis.

Chronic autoimmune thyroiditis has been increasing in prevalence, currently estimated at 0.3 to 1.5 cases per 1,000 individuals, making it the leading cause of hypothyroidism in iodine-sufficient regions [11]. The present study found a higher incidence of autoimmune thyroid disorders than previously reported in the literature. While the role of vitamin D in calcium homeostasis and bone metabolism is well established [12], its involvement in autoimmune thyroid disease remains an area of ongoing investigation. A 2013 meta-analysis by Feng M et al. highlighted a potential association between vitamin D receptor gene polymorphisms and autoimmune thyroiditis, implicating vitamin D signaling in the modulation of pro-inflammatory immune responses [13]. Several clinical studies have also reported an inverse correlation between serum 25(OH)D levels and the presence of thyroiditis or hypothyroidism; however, many of these investigations have primarily focused on thyroid hormone levels rather than the presence or titers of thyroid-specific autoantibodies [14,15].

In the present study, a negative correlation was established between anti-TPO levels and 25(OH)D levels, reaffirming previous qualitative findings in the literature. Notably, 49% of hypothyroid cases in the study exhibited vitamin D deficiency, compared to the international prevalence of 51.8% in hypothyroid individuals, significantly higher than the 39% prevalence observed in the general population, as reported by the Endocrine Society of the United States [16]. Most cases in the study involved female patients, particularly those with hypothyroidism, aligning with existing literature [12-16].

Several studies have shown that reduced free T4 levels may serve as potential predictors of vitamin D deficiency in patients with HT, underscoring the hormone’s possible immunomodulatory role [15-17]. The therapeutic impact of vitamin D supplementation in autoimmune thyroiditis remains an active area of investigation. In contrast to our findings, a meta-analysis by Jiang H et al. reported that vitamin D supplementation increased serum 25(OH)D levels and modified anti-TPO antibody levels, without a significant association with thyroid function parameters [18]. Krysiak R et al. demonstrated that vitamin D supplementation led to a significant reduction in anti-TPO antibody titers and an improvement in free T4 levels and overall thyroid function, suggesting a beneficial effect on autoimmune activity [19].

The primary limitations of this study include the sample size, the detection criteria for HT cases, and the availability of laboratory tests for monitoring anti-TPO and 25(OH)D levels. These limitations underscore the need for larger, multicenter studies to establish optimal vitamin D thresholds and assess its therapeutic potential in autoimmune thyroiditis.

## Conclusions

This study confirms an inverse correlation between vitamin D deficiency and chronic autoimmune thyroiditis, as indicated by elevated anti-TPO levels in patients with lower 25(OH)D concentrations. The findings support vitamin D’s immunomodulatory role in thyroid autoimmunity and its potential impact on disease progression. Despite its retrospective design and sample size limitations, the observed negative correlation between vitamin D and TSH, along with trends in free T4 levels, underscores the need for further investigation. Future multicenter studies should aim to establish precise vitamin D thresholds in autoimmune thyroid disease and evaluate the therapeutic potential of vitamin D supplementation in disease modulation.
